# Fine mapping a quantitative trait locus underlying seedling resistance to gummy stem blight using a residual heterozygous lines-derived strategy in cucumber

**DOI:** 10.3389/fpls.2022.968811

**Published:** 2022-09-02

**Authors:** Jianan Han, Shaoyun Dong, Xiaoping Liu, Yanxia Shi, Diane M. Beckles, Xingfang Gu, Han Miao, Shengping Zhang

**Affiliations:** ^1^Institute of Vegetables and Flowers, Chinese Academy of Agricultural Sciences, Beijing, China; ^2^Department of Plant Sciences, University of California, Davis, Davis, CA, United States

**Keywords:** *Cucumis sativus* L., gummy stem blight, residual heterozygous line (RHL)-derived strategy, fine-mapping, candidate genes

## Abstract

Gummy stem blight (GSB), caused by *Didymella bryoniae*, is one of the most devastating diseases that severely reduces cucumber production. Developing resistant varieties would be an effective strategy to control GSB. Although several GSB-resistant QTLs have been reported, causal genes for GSB resistance have not yet been identified in cucumber. A novel loci *gsb3.1* for seedling GSB resistance from the “PI 183967” genotype was previously identified in a 1.7-Mb interval on chromosome 3. In this study, we developed a residual heterozygous line-derived strategy from Recombinant Inbred Lines to perform fine mapping, and with this approach, the *gsb3.1* locus was narrowed to a 38 kb interval. There were six predicted genes at the *gsb3.1* locus, four of which differed in expression in the GSB-resistant compared to the susceptible lines after fungal inoculation. These candidate genes (*Csa3G020050*, *Csa3G020060*, *Csa3G020090*, and *Csa3G020590*) within the *gsb3.1* locus could be helpful for the genetic study of GSB resistance and marker-assisted selection in cucumber. Phylogenetic analyses indicated that the resistant *gsb3.1* allele may uniquely exist in the wild species present in the Indian group, and that nucleotide diversity was significantly reduced in cultivated accessions. Therefore, the *gsb3.1* allele could be introgressed into existing commercial cultivars and combined with other resistance QTLs to provide broad-spectrum and robust GSB resistance in cucumber.

## Introduction

Breeding for disease resistance in vegetable crops is critical to maintain yield and quality. Cucumber (*Cucumis sativus* L.) is one of the most important vegetables. However, cucumber production in greenhouse is greatly threatened by Gummy Stem Blight (GSB), caused by *Didymella bryoniae* (Kothera et al., 2003; Li, 2007). *Didymella bryoniae* often cause death at the seedling stage, and yield losses and quality decline at the adult stage (Liu et al., 2017). Exploiting and utilizing resistant resources is the most important and effect method to control GSB in cucumber. Some GSB-resistant resources have been identified (Meer et al., 1978; Wyszogrodzka et al., 1986; Wehner and Amand, 1993; Chen et al., 1995; Wehner and Shetty, 2000; Li, 2007; Lou et al., 2013), including wild cucumber (*Cucumis sativus* var. *hardwickii*; Liu et al., 2017; Zhang et al., 2017) and the wild perennial congener of cucumber (*Cucumis hystrix* Chakr.; Chen et al., 1995). However, most of the resistant germplasm have low resource utilization. In our previous study, “PI 183967” was shown to possess GSB resistance both in seedlings and in adult stems (Liu et al., 2017; Zhang et al., 2017). “PI 183967” belongs to *C. sativus* var. *hardwickii* and is considered as the wild progenitor of cultivated cucumber (Qi et al., 2013; Li et al., 2022). Therefore, identifying GSB-resistant alleles in “PI 183967” and transferring them to existing cucumber cultivars for breeding is urgently needed.

Genetic resistance to disease in plants can be classified as showing “adult-plant resistance” or “seedling resistance” (Barcellos et al., 2000; Zhou et al., 2022). Adult-plant resistance is manifested in mature plants, while seedling resistance is durable at all developmental stages (Riaz et al., 2016). In cucumber, GSB resistance at the adult-plant stage is seen on stems and at the seedling stage on leaves. Different conclusions have been drawn about a potential correlation between GSB resistance at the seedling and adult stage. Li (2007) assessed GSB resistance in different varieties and found that there was a significant correlation of GSB resistance in seedlings and adult stems. However, Liu (2013) founded that there was no correlation for GSB resistance between seedling and stem using a diallel design. Therefore, GSB resistance in seedlings may be independent of resistance in adult stems. Due to variable and heterogeneous environments in the field, and the long identification period when studying GSB at the adult stage, most studies for GSB resistance are focused on seedlings, and have been advanced such that several preliminary QTL studies have been conducted. In “PI 183967,” six QTLs, *gsb3.1*, *gsb3.2*, *gsb3.3*, *gsb4.1*, *gsb5.1*, and *gsb6.1*, related to seedling GSB resistance have been identified on Chrs.3, 4, 5, and Chr.6 (Liu et al., 2017), however no candidate genes have been reported.

Positional cloning is a reliable method to identify candidate genes for many agronomic traits controlled by Quantitative Trait Loci (QTL). Many studies carry out fine-mapping to dissect complex quantitative traits using Near Isogenic Lines (NILs), Introgression Lines (ILs), or Chromosome Segment Substitution Lines (CSSLs). These methods are able to effectively reduce the effects from other QTLs. However, constructing these segregating populations by backcross introgression is laborious and time-consuming (Watanabe et al., 2011). Tuinstra et al. (1997) first described an effective strategy using Heterogeneous Inbred Families (HIFs), and identified two QTLs for seed weight in sorghum. Thereafter, this strategy was referred to as the residual heterozygous line (RHL) strategy (Yamanaka et al., 2005), and it has been used for map-based cloning (Watanabe et al., 2009, 2011), including plant disease resistance. RHL are heterozygous at the target locus, but their genetic background is homozygous across the whole genome, which simplifies dissecting multi-loci regions to identify a single locus underlying complex quantitative traits. In soybean, two heterogeneous RILs across flanking markers were used to develop NILs and two genes (*QRfs2* and *QRfs1*) for Sudden Death Syndrome (SDS) were identified (Triwitayakorn et al., 2005). Therefore, utilization of RHLs in fine-mapping is an effective and convenient way to detect candidate genes. Similar to most disease-related traits, GSB resistance at the seedling stage in “PI 183967” is a complex quantitative trait influenced by genetic and environmental effects (Liu et al., 2017).

In our previous study, a QTL *gsb3.1* for seedling GSB resistance from “PI 183967” was identified. To determine genes potentially related to GSB resistance at the seedling stage in “PI 183967,” *gsb3.1* (explaining 7.4% phenotypic variation) was further fine-mapped to a 38 kb interval using a modified RHL strategy. Based on sequence alignments and expression pattern analysis, candidate genes for *gsb3.1* were identified. Our study will accelerate the exploration of genetic mechanisms for GSB resistance and provides a theoretical basis for molecular marker-assisted selection and GSB resistance breeding in cucumber.

## Materials and methods

### Plant materials

A 160 RIL (Recombinant Inbred Line) population derived from “PI 183967” resistant to GSB and “931” the susceptible parent was used for preliminary mapping for GSB resistance (Liu et al., 2017; Zhang et al., 2017). Lines homozygous for *gsb3.1* alleles were selected from this population. According to disease index for GSB resistance at the seedling stage and SLAF-seq data of RILs (Unpublished), a resistant RIL line LM116, and a susceptible RIL line LM34 were selected to develop an F_1_ population in the fall of 2019, as the “Residual heterozygous line” (RHL) for *gsb3.1*. Seeds from the F_1_ were sown in the spring of 2020 to construct segregating populations (F_2_) for fine mapping.1,000 F_2_ plants were used to screen recombinants in the fall of 2020 using flanking markers developed in the previous study, and recombinants were sown in the spring of 2021 to obtain F_2:3_. F_2:3_ plants were grown in the fall of 2021, and used for GSB resistance evaluation. Five plants for each F_2:3_ family were evaluated in one replicate, and all plants were arranged with complete randomized blocks design with three replications at the Institute of Vegetables and Flowers, Chinese Academy of Agricultural Sciences.

### Evaluation of disease index for GSB resistance at the seedling stage

**Figure 1:**
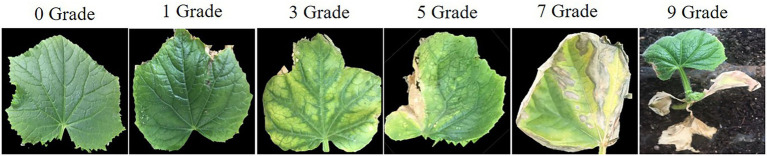
The disease rating scale of leaves at the seedling stage after infection with *Didymella bryoniae*.

The specific strain *Ascochyta citrullina of Didymella bryoniae* was obtained from the Vegetable Disease Prevention and Control Innovation Team (Institute of Vegetables and Flowers, Chinese Academy of Agricultural Sciences). A suspension at 10^6^ spores/ml was used for inoculation. All plants used for inoculation were grown in the greenhouse, and the prepared spore suspension sprayed onto all leaves when the second true leaf was fully opened. Relative humidity was maintained at 80–100%, temperatures ranged from 20°C to 23°C, with 16 h light/8 h dark as the light regime. The disease grade of each leaf was assessed at 7 to 10 days after inoculation as described previously (Liu et al., 2017): Grade 0 = no GSB spots, Grade 1 = 0%–5% GSB spots, Grade 3 = 6%–25% GSB spots, Grade 5 = 26%–50% GSB spots, Grade 7 = 51%–75% GSB spots, and Grade 9 = 76%–100% GSB spots, or the whole seedling dead (Figure 1). GSB resistance of each plant was calculated according to the following Disease Index (DI):


$$\begin{array}{l} DI\;\left(\%\right)\\ =100\times \frac{\sum \;\left(\mathrm{Number of plants with disease rating}\times \mathrm{Disease rating}\right)}{\mathrm{Highest disease rating}\times \mathrm{Total number of plants}} \end{array}$$

### DNA Extraction and genotyping

Following a modified cetyltrimethylammonium bromide (CTAB) method (Saghai-Maroof, 1984), genomic DNA was extracted from the leaves of the F_2_ plants and two parents, and used for PCR amplification and sequencing. All SSR markers used in this study were developed by Liu et al. (2017). Based on the cucumber (Chinese Long) genome v2 and SLAF-seq data of “LM116” and “LM34” (unpublished), SNP and InDel markers were designed to genotype the F_2_ recombinants for fine-mapping using Primer 3.0 online software^1^ (Supplementary Table S1). The PCR system (20 μl) contained 2 μl of genomic DNA (50 ng/μl), 10 μl 2 × Phanta Max Master Mix, 1 μl forward primer and reverse primer (2 μM), and 6 μl ddH_2_O, followed by the PCR amplification procedure: 95°C for 3 min; 35 cycles of 95°C for 15 s, 58°C for 15 s and 72°C for 1 min, and a final 72°C for 5 min. PCR products were separated by 10% (w/v) non-denaturing polyacrylamide gel for SSR and InDel markers, and then visualized by silver-staining. For SNP markers, PCR products were detected by 1% (w/v) agarose gel electrophoresis and then sequenced (Sangon Biotech, Shanghai, China).

### Linkage map development and QTL mapping

To reduce the *gsb3.1* interval, six markers within the preliminary region flanked by *SSR02451 and SSR07456* were selected based on the SLAF-seq data of RILs derived from “PI 183967” and “931” (unpublished). These six markers were used to construct a genetic linkage map and to perform QTL mapping using the publicly available QTL IciMapping v.3.1 software (Supplementary Table S2). The QTL analysis for GSB resistance was conducted using the DI of RILs at the seedling stage and the Inclusive Composite Interval Mapping (ICIM) program in QTL IciMapping v.3.1.

### Fine mapping of *gsb3.1*

For fine-mapping, two homozygous lines, “LM116” and “LM34,” were selected to construct a similar RHL for *gsb3.1*. The recombinants were selected from 1,000 F_2_ individuals using flanking markers *SSR02451* and *SSR07456*. The progenies from these recombinants were used to evaluate GSB resistance at the seedling stage. For refine the target region of *gsb3.1*, further polymorphic markers (Supplementary Table S2) from SLAF-seq data were used to identify the genotype of recombinants. Information of candidate genes within the defined intervals were obtained from cucumber reference genome 9930_v2.^2^

### Sequence analysis and RT-PCR analysis of annotated genes

The DNA sequences of the candidate genes were identified from the parents “LM116” and “LM34” using specific primers (Supplementary Table S3), and the predicted functions were identified using data from the Cucumber (Chinese Long) genome v2 and the TAIR protein database.^3^

In our pre-experiment, there were no significant expression changes between 12 and 24 h post inoculation (hpi; data not shown), therefore the expression patterns of candidate genes 0, 12, 48, and 96 hpi were identified in the parents used to generate the F_1_ mapping population, i.e., “PI 183967” and “931.” The two parents were grown and inoculated with *Didymella bryoniae* in the greenhouse, after the second true leaf fully opened. All true leaves were collected at 0, 12, 48, and 96 hpi, and stored at −80°C. Total RNA was extracted following the manufacturer’s instruction of TransZol Up Plus RNA Kit (TransGen Biotech), and then first-strand cDNAs were prepared by using TransScript First-Strand cDNA Synthesis SuperMix Kit (TransGen, China). Real-time quantitative RT-PCR was performed using SYBR green Super Mix and CFX96 Real-Time PCR Detection System (Bio-Rad, Hercules, CA, United States). Primers for qRT-PCR were designed using Primer3.0^4^ (Supplementary Table S4). *CsActin* (Csa2G301530) was used as an internal control. All experiments were conducted using three biological repeats. The relative expression levels were calculated following the 2^−ΔΔCt^ method (Pfaffl, 2001).

### Phylogenetic analysis

A panel of 113 cucumber germplasm, including 39 East Asian groups, 19 Xishuangbanna groups, 26 European groups, and 29 Indian groups (Qi et al., 2013) were used to analyze the origin and evolution of the *gsb3.1* region. A total of 333 SNPs in the delimited 38 kb region were used to build the phylogenetic tree using the neighbor-joining algorithm in PowerMarker software, and the iTOL online software^5^ was used to visualize the phylogenetic results. To determine how the *gsb3.1* locus was affected by domestication, we calculated the genetic diversity (π) using vcftools with a step size of 5 kb in the East Asian, Xishuangbanna, European, and Indian groups, respectively.

## Results

### Preliminary mapping of the *gsb3.1* locus

**Figure 2:**
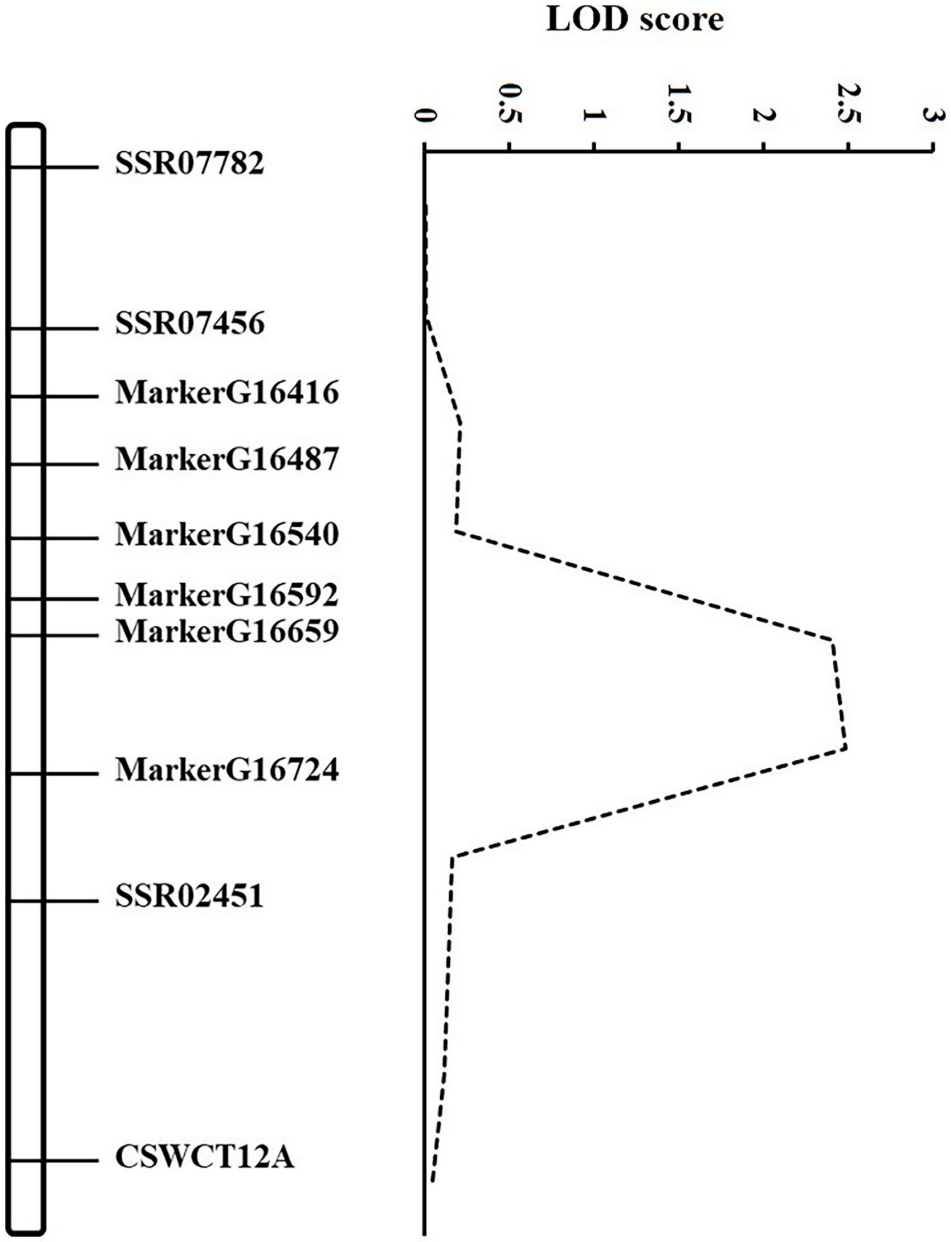
Genetic map of *gsb3.1* on Chromosome 3. Mapping of the *gsb3.1* QTL associated with GSB resistance at the seedling stage of cucumber. The dashed line curve indicates the LOD score relative to the physical position of genetic markers.

According to the GSB disease index of RILs in our previous study (Liu et al., 2017), the DI in the RIL population showed a continuous and normal distribution. Based on the high-density map of SLAF-seq (unpublished), six markers which were evenly distributed across the 1.3 Mb region between *SSR02451* and *SSR07456* were selected. Combined with DI of GSB resistance at the seedling stage in RILs, QTL mapping was conducted using the six novel and three SSR markers (Supplementary Table S1). The *gsb3.1* region was narrowed from 1.3 to 0.7 Mb between *MarkerG16540* (1,800,569 bp) and SSR02451 (2,552,436 bp) based on Cucumber (Chinese Long) genome v2, explaining 7.40% of the phenotypic variance (Figure 2).

### Development of a derived-RHL for *gsb3.1*

**Figure 3:**
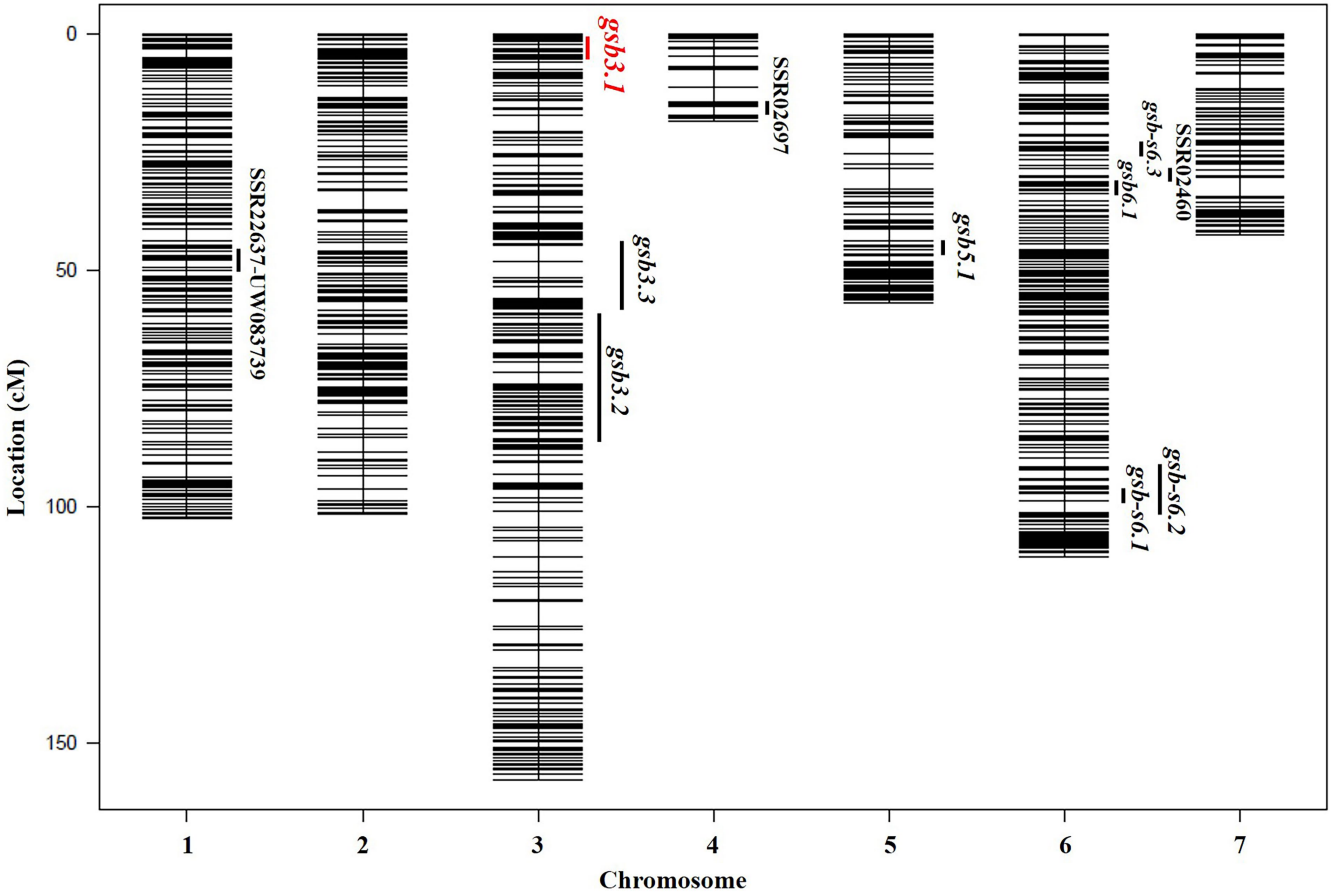
Genetic map and distribution of reported QTLs for GSB resistance at the seedling stage in cucumber. The *gsb3.1* QTL in this study is indicated in red.

According to the genotypes of SSR markers used for preliminary mapping and the SLAF-seq data, none of the individuals from the 160 RILs were heterozygous around the *gsb3.1* locus. To efficiently construct a segregating population, the SLAF-seq data of the 160 RILs were used to analyze genome-wide genotypes. The GSB disease index was assessed in “LM116” and “LM34” which harbored a resistant and susceptible locus for *gsb3.1* respectively, but had identical genetic backgrounds to eliminate interference from other GSB-resistant QTLs (Figure 3). The GSB DI for “LM116” and “LM34” were 35.1 (susceptible) and 65.6 (resistant), respectively, and agreed with data in our previous study. Therefore, these two lines were selected to construct “derived-RHL” for further fine-mapping of the *gsb3.1* locus.

### Fine mapping of *gsb3.1*

**Figure 4:**
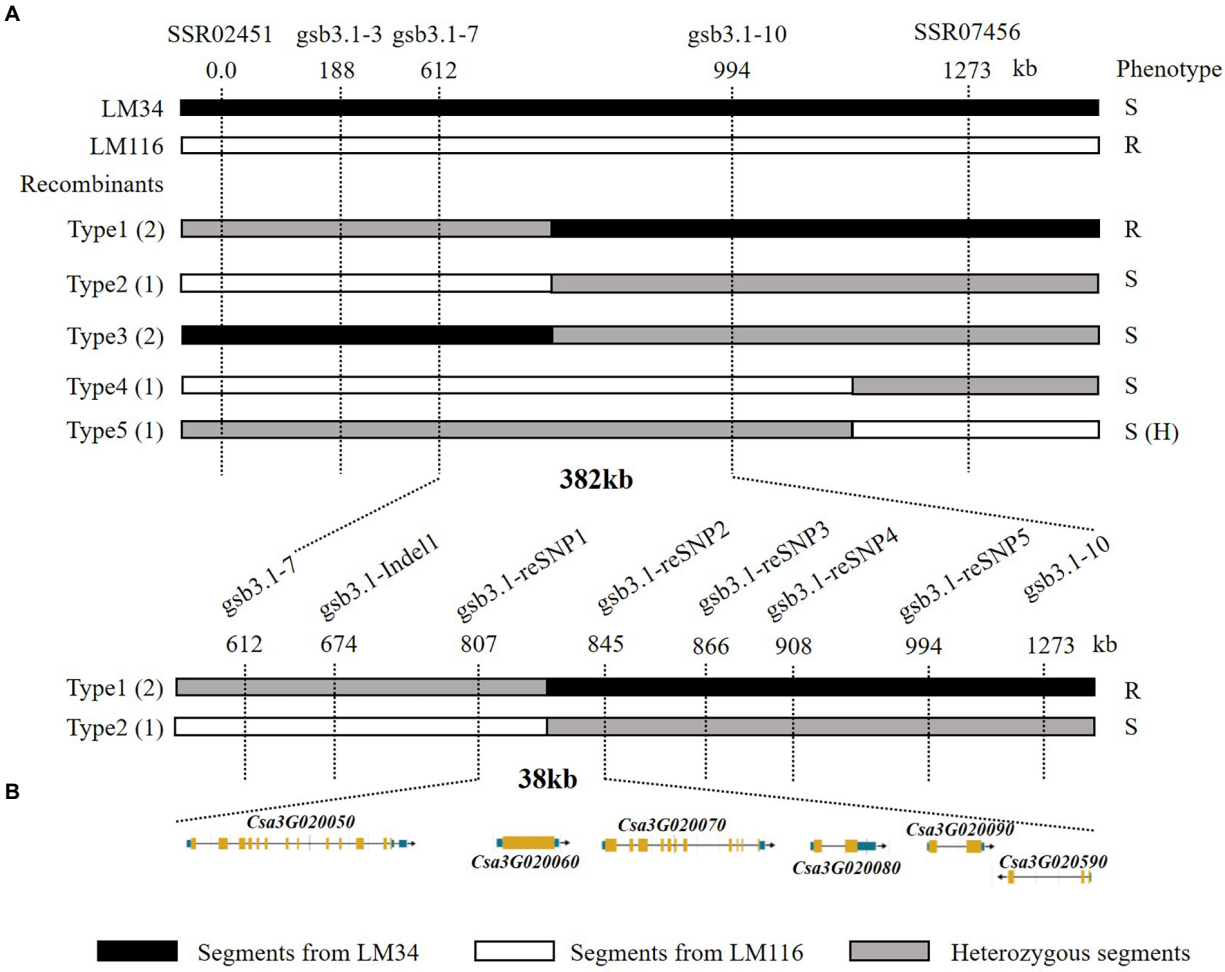
Fine mapping of the *gsb3.1* QTL in cucumber. **(A)** Genetic mapping of *gsb3.1*, using key recombinants from the F_2_ segregated population, i.e., “LM34” and “LM116.” The 38 kb region of *gsb3.1* was narrowed in two phases of fine-mapping. The numbers below the markers indicate the physical position of markers. **(B)** Six annotated genes located in the 38 kb region according to the cucumber “9930”_v2 reference genome. R, resistant; S, susceptible; S (H), susceptible (dominant heterozygous).

A total of 1,000 F_2_ plants derived from “LM116” and “LM34” were used for fine mapping. Two flanking markers *SSR02451* and *SSR07456* were used for preliminary screening. One hundred and twenty-seven recombinants from all F_2_ individuals were further genotyped and used to refine the targeted region. Three InDel markers (*gsb3.1–3*, *gsb3.1–7*, and *gsb3.1–10*) were developed to genotype all these recombinants, and recombinants were classified into five types (Figure 4A). The progenies of all types of recombinants were inoculated and the GSB DI was assessed at the seedling stage. The *gsb3.1* locus was restricted in a 382 kb interval flanked by *gsb3.1–7* (chr3: 1,890,949 bp) and *gsb3.1–10* (chr3: 2,272,924 bp). One InDel and five SNP markers (Supplementary Table S2) were used to screen the recombinants, and *gsb3.1* was finally delimited to a 38 kb interval between *gsb3.1-reSNP1* (chr3: 2,086,270 bp) and *gsb3.1-reSNP2* (chr3: 2,123,799 bp; Figure 4A). According to the cucumber “9930”_v2 reference genome, there were six predicted genes in the *gsb3.1* interval between *gsb3.1-reSNP1* and *gsb3.1-reSNP2* (Figure 4B; Table 1).

**Table 1:** The six predicted genes in the 38 kb fine mapped interval of *gsb3.1* on Chr.3 using the cucumber “9930”_v2 reference genome.

Gene	Position (bp)	Functional annotation
*Csa3G020050*	2,083,620–2,090,586	Chaperone protein DnaJ
*Csa3G020060*	2,103,223–2,104,961	Aspartic proteinase
*Csa3G020070*	2,110,495–2,115,029	Sec14 cytosolic factor
*Csa3G020080*	2,117,181–2,119,000	Heat-shock protein
*Csa3G020090*	2,120,395–2,122,000	Heat-shock protein
*Csa3G020590*	2,122,336–2,124,652	Heavy metal-associated domain

### Sequence analysis of the candidate genes

**Figure 5:**
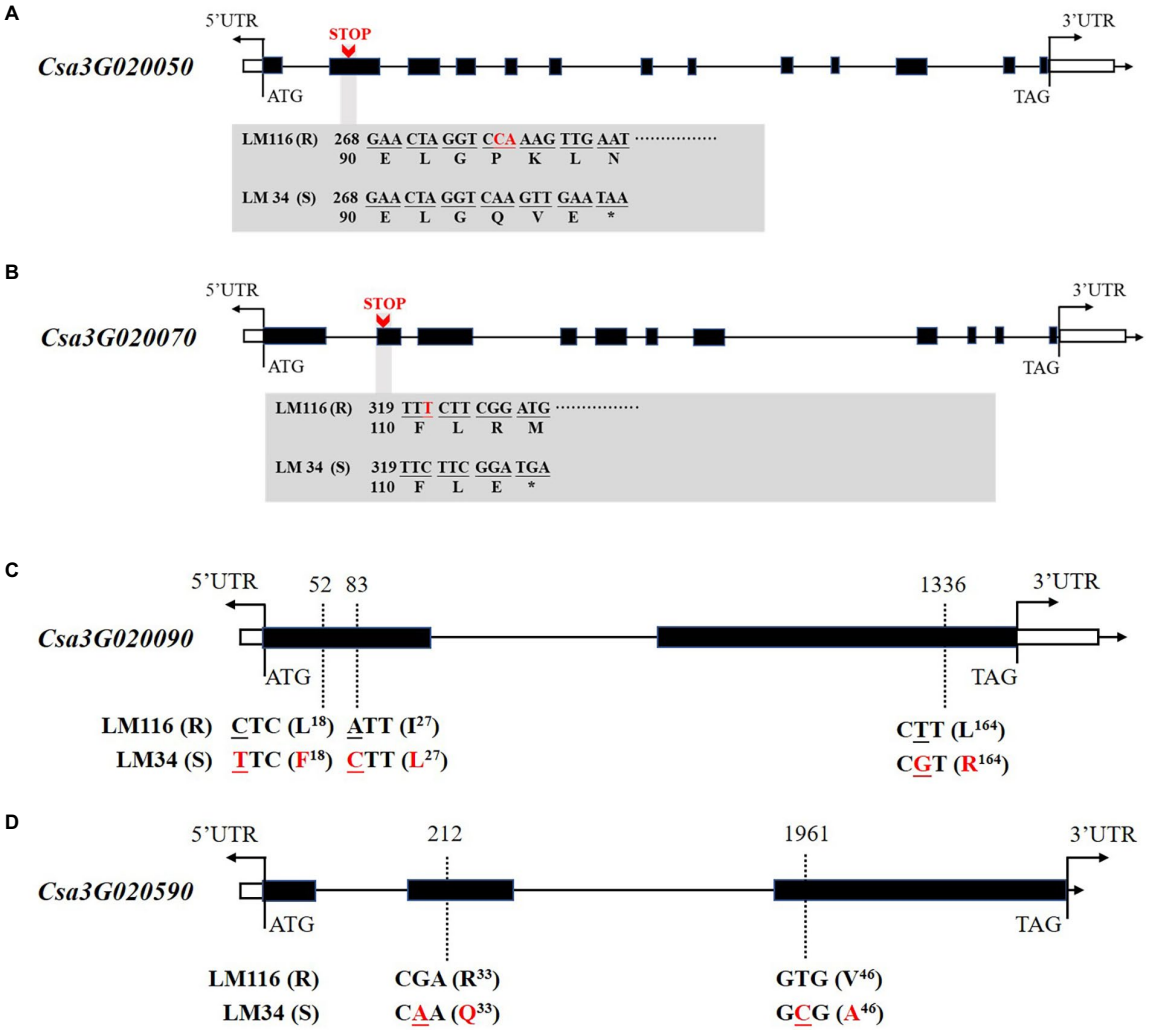
Gene structures and nonsynonymous variations of *Csa3G020050* **(A)**, *Csa3G020070* **(B)**, *Csa3G020090* **(C)**, and *Csa3G020590* **(D)** between LM116 and LM34. Nonsynonymous variations are labeled in red font. “STOP” indicates premature translation termination codons.

The six genes were cloned to identify sequence polymorphisms between the two parents. Four genes harbored nonsynonymous mutations between two genotypes including *Csa3G020050*, *Csa3G020070*, *Csa3G020090,* and *Csa3G020590. Csa3G020050* encodes Chaperone protein DnaJ. In the susceptible line “LM34” a 2 bp deletion at nucleotide 278 in the second exon is predicted to result in a premature translation termination codon, encoding a truncated polypeptide of 96 amino acids (Figure 5A). *Csa3G020070* encodes Sec14 cytosolic factor. In the second exon of the susceptible line “LM34” a 1 bp deletion at nucleotide 321 should also result in a premature translation termination codon, creating a truncated polypeptide of 109 amino acids (Figure 5B). *Csa3G020090* has three non-synonymous SNPs in the coding region between the two parents. A leucine to phenylalanine substitution at amino acid 18, an isoleucine to leucine substitution at amino acid 27, and a leucine to arginine substitution at amino acid 164 are predicted (Figure 5C). *Csa3G020590* harbors two non-synonymous SNPs that leads to an arginine to glutamine substitution at position 33 and a valine to arginine substitution at position 46 (Figure 5D).

### Expression pattern analysis of candidate genes

**Figure 6:**
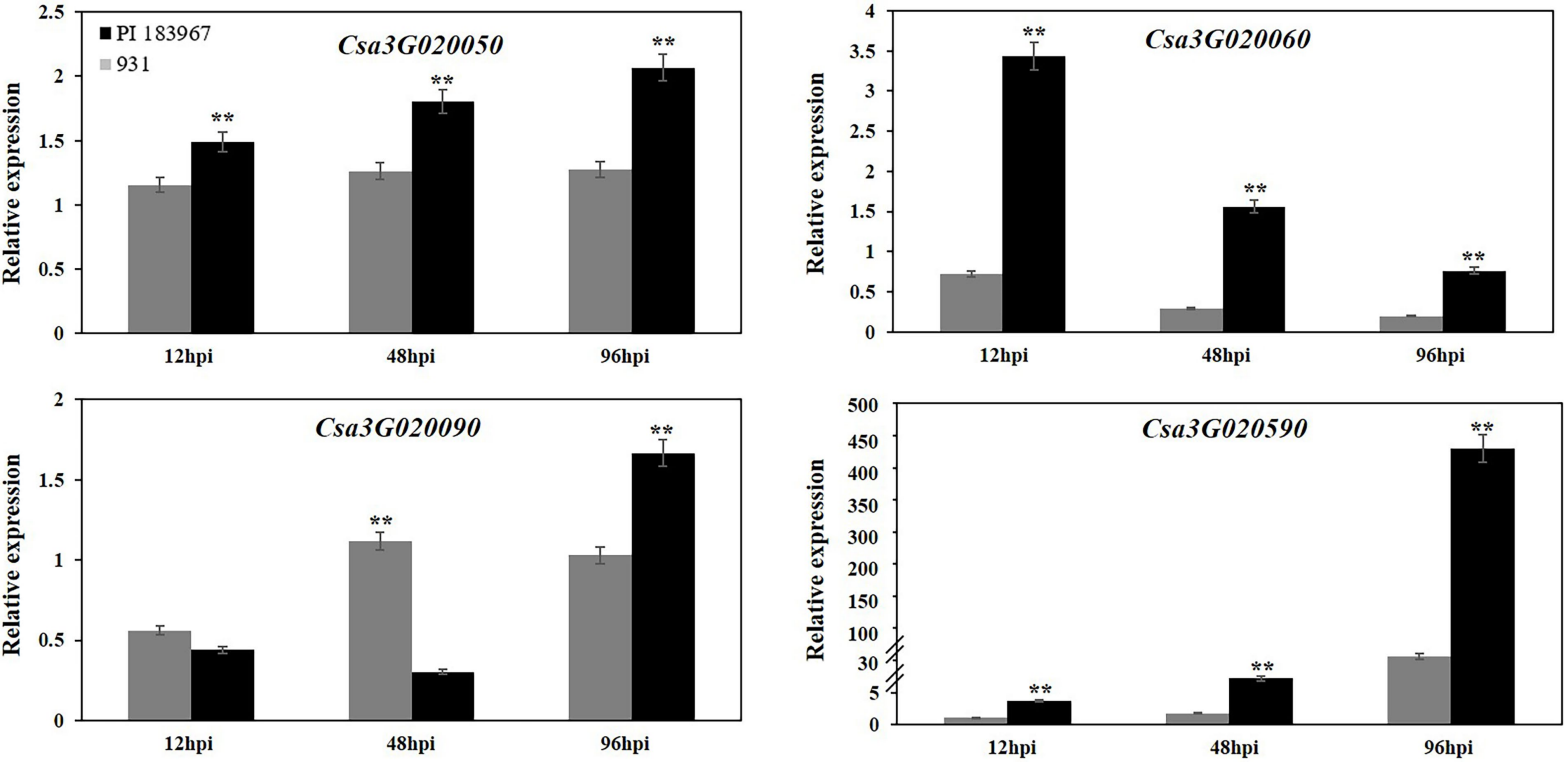
Relative expression levels of candidate genes in “PI 183967” and “931” after inoculation with at 12, 48, and 96 hpi. The Y-axis represents the relative expression level of candidate genes at 12, 48, and 96 hpi, compared with 0 hpi. Asterisks indicate significant differences as determined by ANOVA (^**^p < 0.01).

To determine if the sequence polymorphisms in the *gsb3.1* candidate genes might lead to functional differences, their expression patterns in seedlings before, and up to 96 h post inoculation were analyzed by qRT-PCR. *Csa3G020070* (Sec14 cytosolic factor) and *Csa3G020080* (Heat-shock protein) had very low expression levels at all time points and the data were excluded from the analysis. Of the genes shown in Figure 6*, Csa3G020050* (Chaperone protein DnaJ) expression was unaffected in the GSB-susceptible parent “931,” but was upregulated after inoculation in the GSB-resistant parent “PI 183967.” Compared to 0 hpi, *Csa3G020060* (Aspartic proteinase) was significantly down-regulated after inoculation in “931,” but was upregulated 3-fold in “PI 183967,” however expression decreased steadily in both genotypes over time, but expression was consistently higher in “PI 183967.” *Csa3G020090* expression was down-regulated at 12 hpi in both two parents, but thereafter, gradually returned to normal levels in “931,” but showed increasing expression at 96 hpi in “PI 183967.” Transcripts for *Csa3G020590* were rapidly up-regulated after inoculation in both two parents, but expression in “PI 183967” increased more than 400-fold compared to 100-fold in “931” after 96 hpi. We excluded *Csa3G020070* and *Csa3G020080* as candidates for *gsb3.1* because of their extremely low expression levels. Combined with sequence analysis, we considered *Csa3G020050*, *Csa3G020060*, *Csa3G020090*, and *Csa3G020590* as candidate genes for *gsb3.1* related to GSB resistance in our study.

### Comparative analysis of candidate genes in cucumber germplasms

**Figure 7:**
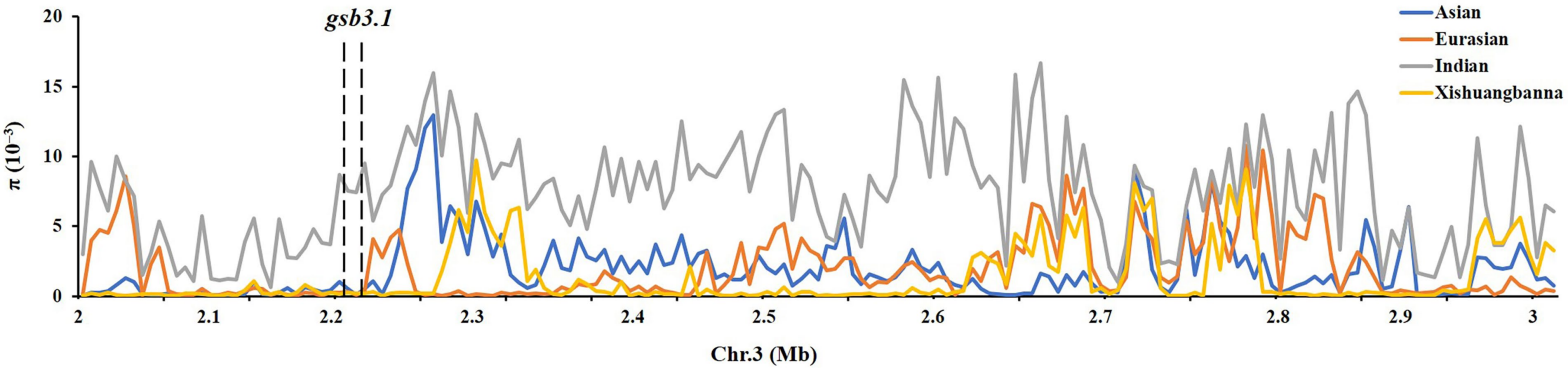
Distribution of nucleotide diversity (π) of four geographic groups. The region of *gsb3.1* on Chr. Three overlaps with a large domestication sweep region showing reduced.

**Figure 8:**
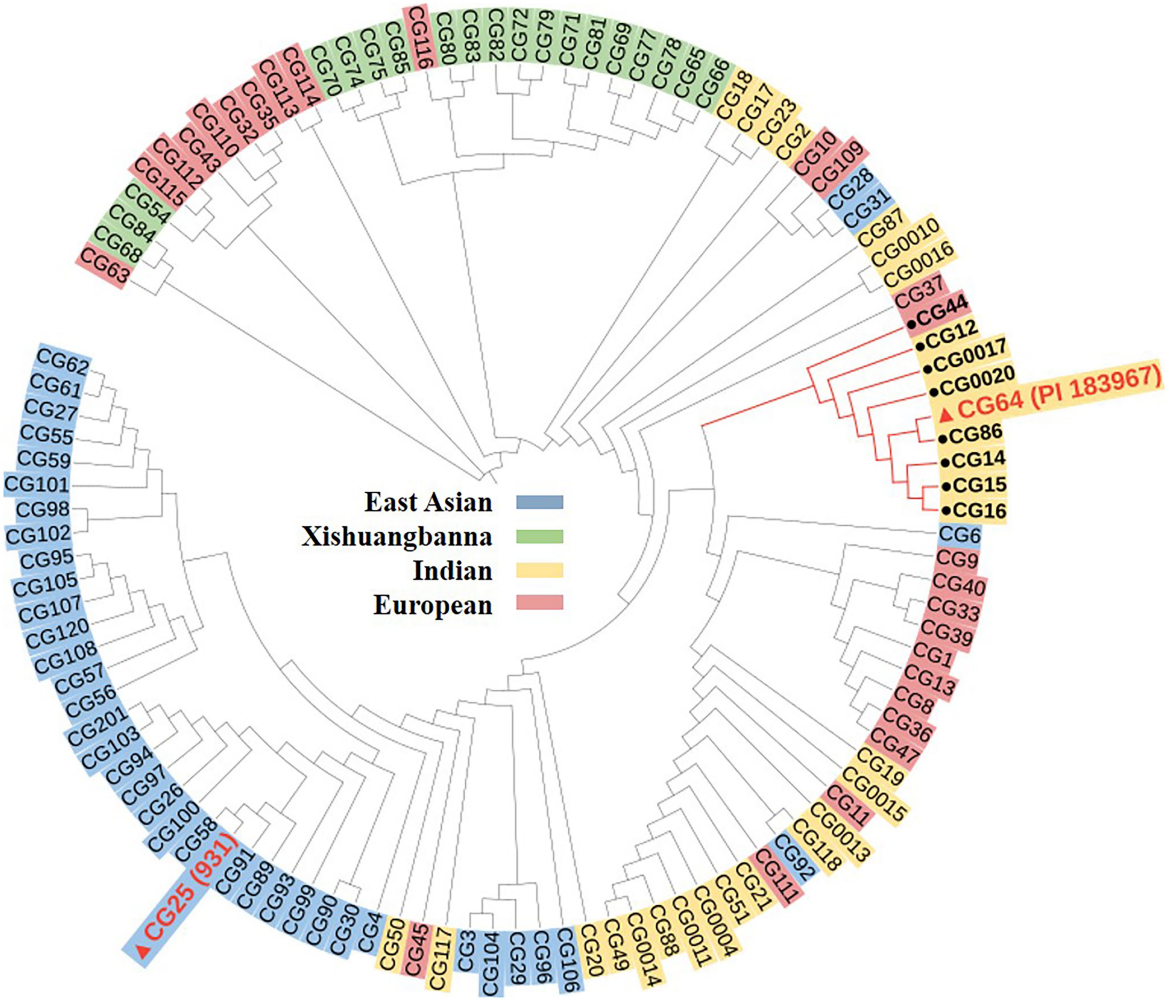
Phylogenetic analysis for *gsb3.1* locus. A dendrogram of 113 cucumber accessions analyzed using 333 SNPs derived from Qi et al. (2013) within the delimited 38-kb *gsb3.1* region.

We then detected nucleotide diversity and the distribution of *gsb3.1* alleles in cucumber accessions from different geographical groups, based on the cucumber genomic variation map (Qi et al., 2013). Scans of the *gsb3.1* genomic region showed significant reductions in nucleotide diversity in the three cucumber cultivated groups compared to the group from India, which is widely believed to be an important center of ancestral cucumber (Qi et al., 2013; Figure 7). A phylogenetic tree was constructed using 333 SNPs within the 38 kb interval. The distribution of *gsb3.1* alleles among the four geographic groups, i.e., the Indian, Xishuangbanna, East Asian, and Eurasian groups, showed that, “PI 183967” (CG64) as the source of the resistant allele at the *gsb3.1* locus, clustered with seven accessions (CG12, CG14, CG15, CG16, CG0017, CG0020, and CG86) from the Indian group and with one accession from the European group (CG44). Of these accessions, CG86 (LJ 90430) was previously identified as having GSB resistance (Wehner and Shetty, 2000). In contrast, the GSB-susceptible “931” (CG25) genotype clustered with most of the East Asian accessions (Figure 8). These results suggest that the *gsb3.1-*resistant allele might have originated from wild species within the Indian group, and due to its absence in most cultivars, this unique locus could be introduced into elite cucumber varieties to increase genetic diversity for resistance to GSB.

## Discussion

### QTLs for GSB resistance at the seedling stage in cucumber

In previous studies, QTLs for GSB resistance at the seedling stage from the *C. hystrix*-resistant genotype were identified on Chrs. 4 and 6 using ILs (Lou et al., 2013), and on Chr. 1, using RILs (Zhang et al., 2018). In *C. sativus var. hardwickii* (“PI 183967”), six QTLs for GSB resistance at the seedling stage were detected on Chrs. 3, 4, 5, and 6 (Liu et al., 2017). Among these QTLs, no overlapping loci were found in populations derived from *C. hystrix and C. sativus* var. *hardwickii* (Figure 3). We therefore speculate that different resistant germplasm resources may carry distinct GSB-resistant genes. To identify the GSB-resistant genes in “PI 183967,” it would be necessary to fine-map the major QTL *gsb5.1* on Chr. 5, but the presence of a chromosome inversion at *gsb5.1* complicates this effort (Yang et al., 2012). Based on previous QTL mapping studies, GSB resistance in “PI 183967” is likely to be controlled by multiple loci, making it possible that several QTLs or genes cooperate to engender GSB resistance. We therefore selected the *gsb3.1* locus for fine-mapping; even though the *gsb3.1* locus did not show high phenotypic variation, it could still provide strong and stable GSB resistance if used in a QTL-pyramiding program for cucumber breeding.

By adding markers between the existing flanking *SSR02451* and *SSR07456* markers, the preliminary interval of *gsb3.1* was reduced to a region of 0.7 Mb. This strategy allowed us to implement precise fine-mapping and to identify candidate gene for *gsb3.1.* However, *gsb3.1* explains only 7.40% of the phenotypic variance for GSB resistance at the seedling stage, therefore, more precise methods to validate these genes will be needed, such as a segregating population derived from backcrossing to the susceptible parent or residual heterozygous lines.

### RHL-derived map-based cloning strategy

The most common approach to identify the responsible gene for a QTL, is to use Near Isogenic Lines (NILs; Dorweiler et al., 1993). However, the backcrossing to the recurrent parent needed to develop NILs is labor intensive and time-consuming. Tuinstra et al. (1997) firstly adopted an improved procedure called Heterogeneous Inbred Family (HIF) analysis to generate NILs that segregate for a specific QTL. This strategy could reduce the effects resulting from other non-causal loci, and facilitate QTL mapping. This strategy has been used in many studies and is referred to as the Residual Heterozygous Line (RHL) strategy (Yamanaka et al., 2005). In our study, we attempted to use the same strategy, but were unable to detect a RHL from our 160 RILs. To solve this problem, we used an alternative procedure for developing RHLs. Two lines with significantly different GSB-resistance at the seedling stage among the RILs were selected to generate “RHL,” which have a different segment at the target QTL, but with homozygous backgrounds. Due to the large effect of the major QTL *gsb5.1* and the small effects of the minor QTLs identified in our previous study, this alternative procedure for developing RHLs allowed us to conduct fine-mapping of a minor QTL as a single gene, and to improve the efficiency of map-based cloning for *gsb3.1*. By using a derived RHL strategy, *gsb3.1* was defined to a 38 kb interval contained six genes. This strategy could also be used for identifying other minor QTLs, and facilitate molecular marker-assisted selection to improve disease resistance by multiple resistant QTL pyramiding in cucumber.

### Sequence and expression analysis of genes for *gsb3.1*

Among the six candidate genes, we identified three with chaperone protein functions, (*Csa3G020050*, *Csa3G020080*, and *Csa3G020090*), an aspartic proteinase (*Csa3G020060*), a Sec14 cytosolic factor (*Csa3G020070*), and a polypeptide with a heavy metal-associated domain (*Csa3G020590*). Sequence alignments between resistant and susceptible lines showed non-synonymous variations in four genes, i.e., *Csa3G020050*, *Csa3G020070*, *Csa3G020090*, and *Csa3G020590*.

Expression analysis of the six candidate genes was also conducted to identify potential functional responses to GSB infection. Among the six genes, *Csa3G020050*, a chaperone protein DnaJ, was significantly up-regulated after inoculation of the GSB-resistant line but not in the GSB-susceptible line. *Csa3G020060*, the aspartic proteinase, significantly increased in expression after inoculation of the GSB-resistant line, but expression decreased in the GSB-susceptible line. *Csa3G020090*, a Heat-shock protein, showed different expression levels at 48 hpi and 96 hpi when the two parents were compared, but not at 12 hpi. Compared with 0 hpi, its expression level was down-regulated, but thereafter, gradually increased to pre-inoculation levels in both parents. *Csa3G020590* (a heavy metal-associated domain gene) showed the most dramatic change in expression after inoculation when the two parents were compared, with significantly higher expression in the GSB-resistant line.

### Functional analysis of possible candidate genes

After combined sequence alignment and expression analysis, we considered four genes (*Csa3G020050*, *Csa3G020060*, *Csa3G020090*, and *Csa3G020590*) as candidates for *gsb3.1*. Notably, *Csa3G020050* had an influential protein truncation that distinguished the GSB-resistant from the GSB-susceptible lines. We speculated that the premature translation termination codon and the truncated protein of *Csa3G020050* in “LM34” had a potential effect on GSB resistance in cucumber. *Csa3G020050* encodes a Chaperone protein DnaJ with important functions in protein folding and various physiological regulation (Szabo et al., 1996). The truncated protein in the susceptible line “LM34” resulted in the lack of the J-domain at the N-terminus and all domains at the C-terminal, which might impair various function, including resistance to disease. DnaJ-like proteins have been shown to interact with viruses by mediating the formation of viral multi-protein complexes (Verchot, 2012), including tobacco mosaic virus (Hwang et al., 1998; Hofius et al., 2007; Shimizu et al., 2009; Verchot, 2012), and soybean mosaic virus (Zong et al., 2020). In addition, plant disease resistance (R) proteins require chaperone proteins to form multi-protein complexes for their folding and functioning (Van et al., 2010).

*Csa3G020060* encodes an aspartic proteinase. Aspartic proteinases have many functions in different biological processes, including stress response, senescence and programmed cell death, and protein processing (Timotijevi et al., 2010; Weina et al., 2016). Aspartic proteinases have been reported to be involved in plant disease resistance. In *Arabidopsis*, aspartic proteases functions in disease resistance signaling to virulent *Pseudomonas syringae* through a peptide signal system (Xia et al., 2014). Another predicted aspartyl protease, AED1 regulates systemic immunity as a part of a homeostatic feedback response (Breitenbach et al., 2014). In rice, *OsCDR1* (a predicted aspartate protease) conferred enhanced resistance against bacterial and fungal pathogens both in *Arabidopsis* and rice (Prasad et al., 2009). It would not be surprising if *Csa3G020060* was part of a signaling pathway for resistance to *Didymella bryoniae.*

*Csa3G020090* encodes a HSP20-like chaperone. As ATP-independent chaperones, HSP20s are able to prevent the unfolding and disassembly of proteins, and their subsequent aggregation (Montfort et al., 2001; Ooijen et al., 2010). HSPs chaperone are able to interact with the leucine-rich repeat (LRR) domains of Resistance (R) proteins in plants, and are required for the hypersensitive response (HR; Ooijen et al., 2010). Heat-shock proteins have been indicated in defense against bacterial pathogens in many studies. In *Nicotiana*, a small heat shock protein (HSP17) has a role in HR-independent defenses against *Ralstonia solanacearum* (Maimbo et al., 2007). In tomato, RSI2, small heat shock protein 20, directly interacted with, and stabilized the tomato resistance protein I-2 to activate the hypersensitive response (Ooijen et al., 2010).

*Csa3G020590* contains a heavy metal-associated domain (HMA). Metallochaperones play various roles in plant development and defense responses, including maintaining heavy metal homeostasis and detoxification, transcriptional responses to environmental changes, and plant-pathogen interactions (De Abreu-Neto et al., 2013; Zschiesche et al., 2015). In rice, two MAX (*Magnaporthe oryzae* avirulence and ToxB-like) effectors were recognized by integrated HMA domains in NLRs (nucleotide-binding leucine rich repeat receptors), which directly bind AVR-Pik to activate plant defenses (Guo et al., 2018; Maidment et al., 2021). Mutations in the HMA-binding domain perturbed NLR-binding and affected effector recognition of *M. oryzae* (Guo et al., 2018). In our study, we identified two nonsynonymous SNPs within the HMA domain in *Csa3G020590* when the two parents were compared. Mutations in this domain would interfere with HMA binding of the effectors, altering pathogen recognition in the susceptible cucumber genotype.

In summary, we considered *Csa3G020050*, *Csa3G020060*, *Csa3G020090,* and *Csa3G020590* as candidate genes for *gsb3.1* related to GSB resistance in our study. However, further work is needed to identify the causal genes controlling GSB resistance and their regulatory mechanism still needs to be further verified and researched.

### *gsb3.1* is a unique resistant locus from Indian group

To explore the genetic diversity and distribution of *gsb3.1*, we further analyzed the nucleotide diversity of alleles at the *gsb3.1* locus from different geographic groups among the available cucumber germplasm. We found that the nucleotide diversity of *gsb3.1* was significantly reduced in the cultivated groups compared to the Indian group which contains the wild form *C. sativus var. hardwickii* (Figure 7). We speculate that there was a selective sweep across the genomic region including the *gsb3.1* locus. We then analyzed the distribution of *gsb3.1* in the four genetically similar groups.

Phylogenetic analysis showed that *gsb3.1* existed only in eight Indian accessions and one European accession, and was absent in the East Asian, Xishuangbanna, and most of the European accessions. The accessions in the Eurasian group are native to Europe and the United States, those in the East Asian group are mainly from China, Korea, and Japan, and the accessions in the Xishuangbanna group are mainly from tropical southwestern China. Previous studies found that Eurasian accessions contain several R genes associated with fungal disease resistance on Chr. 2, but these genes are absent in accessions from the East Asian group (Kang et al., 2011), and substantial divergence was detected among the three cultivated groups (Qi et al., 2013). The potential domestication sweeps in the cucumber genome showed that the *gsb3.1* region was in a selective sweep region, and the decreased nucleotide diversity in three cultivated groups (Qi et al., 2013). Our study further confirmed that the Eurasian and Indian groups may possess more disease resistance, as a result of their distinct geographic distributions, and the strong differentiation likely due to adaptation to the local microbial environment (Qi et al., 2013). The resistant *gsb3.1* allele might have originated from the wild progenitor of cultivated cucumber from India, and was under environmental selection. However, *gsb3.1* has lower phenotypic variation, compared with *gsb5.1* and other QTLs in “PI 183967.” Therefore, *gsb3.1* could be introgressed into excellent cultivars *via* marker-assisted selection and be stacked with other resistance QTLs for stronger GSB resistance in cucumber.

## Data availability statement

The original contributions presented in the study are included in the article/Supplementary material, further inquiries can be directed to the corresponding authors.

## Author contributions

SP and XG designed the experiments. JH performed the experiments, analyzed the data, and wrote the manuscript. SD, XL, DB, and SP revised the manuscript. HM participated partial experiments. YS provided *Didymella bryoniae*. All authors contributed to the article and approved the submitted version.

## Funding

This work was supported by Key-Area Research and Development Program of Shandong Province (2021LZGC016), the earmarked fund for Modern Agro-industry Technology Research System (CARS-23), the Agricultural Science and Technology Innovation Program of the Chinese Academy of Agricultural Sciences (CAAS-ASTIP-IVFCAAS), and the Key Laboratory of Biology and Genetic Improvement of Horticultural Crops, Ministry of Agriculture and Rural Affairs, China.

## Conflict of interest

The authors declare that the research was conducted in the absence of any commercial or financial relationships that could be construed as a potential conflict of interest.

## Publisher’s note

All claims expressed in this article are solely those of the authors and do not necessarily represent those of their affiliated organizations, or those of the publisher, the editors and the reviewers. Any product that may be evaluated in this article, or claim that may be made by its manufacturer, is not guaranteed or endorsed by the publisher.

## Footnotes

^1^ https://primer3.ut.ee/

^2^ http://cucurbitgenomics.org/organism/2

^3^ http://www.arabidopsis.org/

^4^ https://primer3.ut.ee/

^5^ https://itol.embl.de/
