# Supplementary material for: Fine mapping a quantitative trait locus underlying seedling resistance to gummy stem blight using a residual heterozygous lines-derived strategy in cucumber
Source: Front Plant Sci. 2022 Sep 2;13:968811. doi: 10.3389/fpls.2022.968811 (PMC9480501; doi:10.3389/fpls.2022.968811)
Supplement: Supplementary file 1 [file Data_Sheet_1.DOCX]

Supplementary Material

# Supplementary Tables

**Table S1 Information of markers within *gsb3.1* used in this study**

| Marker | Physicial position (bp) |
| --- | --- |
| SSR07782 | 277,907 |
| SSR07456 | 1,278,671 |
| MarkerG16416 | 1,452,966 |
| MarkerG16487 | 1,666,218 |
| MarkerG16540 | 1,800,569 |
| MarkerG16592 | 1,945,801 |
| MarkerG16659 | 2,138,262 |
| MarkerG16724 | 2,416,274 |
| SSR02451 | 3,212,336 |
| CSWCT12A | 5,421,428 |

**Table S2 Primers developed for fine mapping of *gsb3.1***

| Primer Name | Position (bp) | Forward primer (5’→3’) | Reverse primer (5’→3’) | Type |
| --- | --- | --- | --- | --- |
| SSR07456 | 1,278,671 | TCATCCGTGGTTTCTCCTTC | TTTTTCTCCCTCCCATCCAT | SSR |
| gsb3.1-3 | 1,466,790 | TTGGTTCGATCGTAAATTTGGC | CACTGCATGCTTAGTTCACTTA | InDel |
| gsb3.1-7 | 1,890,949 | TATGAATCATTGGGTGGGCC | ACAATGGGCATATCTAAAAGGC | InDel |
| gsb3.1-Indel1 | 1,952,870 | ATCCAATGACCGAGTTCAGC | TGCAACTAAATCTTCAGTCGC | InDel |
| gsb3.1-reSNP1 | 2,086,270 | GGAGTTGGCCATTGTTGAT | GGTCACGGAGCATACAAATCA | SNP |
| gsb3.1-reSNP9 | 2,112,947 | AAAAAAAACTTCCAGCC | ATTTGATGACTACGGGCAGC | SNP |
| gsb3.1-reSNP3 | 2,145,302 | TGTTACCAACGTAGAACTTCAG | GCTCTCTCAAATCTTTCAATGC | SNP |
| gsb3.1-reSNP4 | 2,186,914 | TGGCTACCCTCAAGATCAAGA | CAACATCTCGGGACACAACA | SNP |
| gsb3.1-reSNP5 | 2,223,313 | CGAACAACTGATGGAGGGAG | GCTTGCTGAAACTTTGGACA | SNP |
| gsb3.1-10 | 2,272,924 | TCATCCACGCGATCTCCTTA | GAGCTCTGCGATAAGACTGG | InDel |
| SSR02451 | 3,212,336 | TCACCTCCTTCCTCACATCC | GTTCTAGAGGGAGTTCCGGG | SSR |

**Table S3 Primers of candidate genes for sequence analysis**

| Primer name | Forward primer (5’→3’) | Reverse primer (5’→3’) |
| --- | --- | --- |
| Csa3G020050 | TCCATGCTCTCACGTCATTT | TTCACAGTCCACTTCATCCC |
| Csa3G020060 | GAAGAAAGAAGAAAGAGGGTGT | GCAACCACTTCATTCCTTGC |
| Csa3G020070 | GGGCCTCAAACATGGGAAAT | GGCAAGTTTTGTAAGTCTTCGA |
| Csa3G020080 | GACCATGATGATCGTTCTGTTG | ACATTACCTCCGAAACCAGAAT |
| Csa3G020090 | TAGGAGCAGGATCAAGGAGG | TACAGATTCGCAGGCAAGTC |
| Csa3G020590 | ATGAGTTGCAATGGTTGTCG | TCGCAGTTGTTCATTTGGGA |

**Table S4 Primers of candidate genes for qRT-PCR analysis**

| Primer name | Forward primer (5’→3’) | Reverse primer (5’→3’) |
| --- | --- | --- |
| qCsa3G020050 | GCTCAAGTCTATCATCCTGACA | ATTTCGTATGCTTCGCAGATTC |
| qCsa3G020060 | ACTACTTTTACTATGCTCCCGG | CCGGTATTTTCTTCAATCCGTC |
| qCsa3G020070 | ATCAATGCTGGGTCAGGATTTA | TGAATTTTAGCTAACGTGCGAG |
| qCsa3G020080 | GACCATGATGATCGTTCTGTTG | ACATTACCTCCGAAACCAGAAT |
| qCsa3G020090 | TAGGAGCAGGATCAAGGAGG | TACAGATTCGCAGGCAAGTC |
| qCsa3G020590 | ATGAGTTGCAATGGTTGTCG | TCGCAGTTGTTCATTTGGGA |
